# Association of human gut microbiota composition and metabolic functions with *Ficus hirta Vahl* dietary supplementation

**DOI:** 10.1038/s41538-022-00161-3

**Published:** 2022-09-27

**Authors:** Ruiming Xiao, Guangjuan Luo, Wanci Liao, Shuting Chen, Shuangyan Han, Shuli Liang, Ying Lin

**Affiliations:** 1 South China University of Technology South China Univ Technol, School of Biology & Biological Engineering, Guangzhou, China; 2 Guangdong Key Lab Fermentation & Enzyme Engineering, Guangzhou, 510006 China

**Keywords:** Nutrition, Microbiome

## Abstract

*Ficus hirta Vahl* (FHV), a traditional herbal ingredient of the tonic diet, receives increasing popularity in southern China. However, it is largely unknown that how a FHV diet (FHVD) affects the human gut microbiome. In this exploratory study, a total of 43 healthy individuals were randomized into the FHVD (*n* = 25) and Control (*n* = 18) groups to receive diet intervention for 8 weeks. 16S rRNA gene sequencing, metagenomic sequencing and metabolic profile of participants were measured to assess the association between FHV diet and gut microbiome. A preservation effect of *Faecalibacterium* and enrichment of *Dialister*, *Veillonella*, *Clostridium*, and *Lachnospiraceae* were found during the FHVD. Accordingly, the pathway of amino acid synthesis, citrate cycle, coenzyme synthesis, and partial B vitamin synthesis were found to be more abundant in the FHVD. In addition, serine, glutamine, gamma-aminobutyric acid, tryptamine, and short-chain fatty acids (SCFAs) were higher after the FHVD. The conjoint analysis of FHV components and in-vitro fermentation confirmed that the improved SCFAs concentration was collectively contributed by the increasing abundance of key enzyme genes and available substrates. In conclusion, the muti-omics analysis showed that the FHVD optimized the structure of the gut microbial community and its metabolic profile, leading to a healthy tendency, with a small cluster of bacteria driving the variation rather than a single taxon.

## Introduction

A healthy, or tonic diet is to applying functional food into daily diet for healthcare. It has become an emerging lifestyle element with rapidly increasing popularity among a wide range of people in recent decades. *Ficus hirta Vahl* (FHV), also called Wuzhimaotao in southern China, belongs to the Moraceae in the genus Ficus. Its dried root is commonly used in soups and health products in South China. Currently, diverse biochemical components have been identified in FHV, including flavonoids, coumarins, terpenes, alkaloid phenolic acids and glycosides^1,2^. It was confirmed that FHV, as a traditional Chinese medicine, possessed antioxidant and anti-inflammatory activities^3–5^, but most concentrated on its in-vitro functions and performed animal trials.

The FHV diet (FHVD) usually includes FHV in the daily diet in the form of soup, the local residents are used to consumed FHVD for healthcare, especially in hot and damp districts of South China. In parallel with the development of preventive disease treatments, the interaction between personalized diet and host health received wide attention in recent years^6,7^. Beyond medical effects, previous reports have emphasized the importance of disease prevention through a customized diet such as Gluten-free diet^8^.

In the past decade, the development of the human genome project has accelerated the progress of precise nutrition^9^. The gut microbiome, known as the second genome of the human body, can directly interact with ingested nutrients and alter their efficacy, bioavailability and affecting host physiology^10^. More recently, many studies have shown that gut microbiome composition is closely related to dietary habits^11^. For instance, the vegetarian diet increased the abundance of *Bacteroides* and *Faecalibacterium* and decreased the abundance of *Clostridium* cluster XIVa^12^. A commonly known Western diet was verified to reduce the bacteria that metabolize dietary plant polysaccharides such as *Roseburia*, *Eubacterium rectale*, and *Ruminococcus bromii*^13^, suggesting that gut microbial changes were varied based on diet habits and differences in nutrient intake. With the development of omics technology, comparison between Western and Mediterranean populations by metagenomics and metabolomics approaches revealed that diet might have a stronger influence on microbial metabolism than on taxa^14^ in which the microbiota metabolites, such as vitamins, amino acids, and short-chain fatty acids (SCFAs), are considered to benefit human health^15^. It is known there is an important link between diet and the gut microbiome, but it is largely unknown how the FHVD affects the human gut microbial composition and metabolic function.

In this exploratory research, we utilized next-generation 16S rRNA gene sequencing, shotgun metagenomic sequencing and widely targeted metabolomics to perform a combinatorial analysis of the effect of FHVD on the microbial taxonomy, functions, and metabolic profiles. This study offers comprehensive insights into the interaction between the FHV tonic diet and gut microbiota.

## Results

### Nutrition intake during intervention

To reduce the impact of daily dietary intake on gut microbiota, all participants were required to repast at a specific dining hall and record their food for calculating the daily intake of energy, protein, fat, and carbohydrate at the sampling weeks (week 0 and week 8), for which there were no significant differences (*P* > 0.05) in micronutrient intake (Supplementary Table 3), while the intake of FHV roots in two treatment groups was distinctly different (*P* < 0.001), as presented in Supplementary Table 1. Besides, a preliminary experiment of FHVD intervention was conducted within 9 individuals. However, little significant difference of blood biochemicals was found as presented in Supplementary Table 4.

### Gut microbial taxonomy alteration in the *Ficus hirta Vahl* diet

**Fig. 1:**
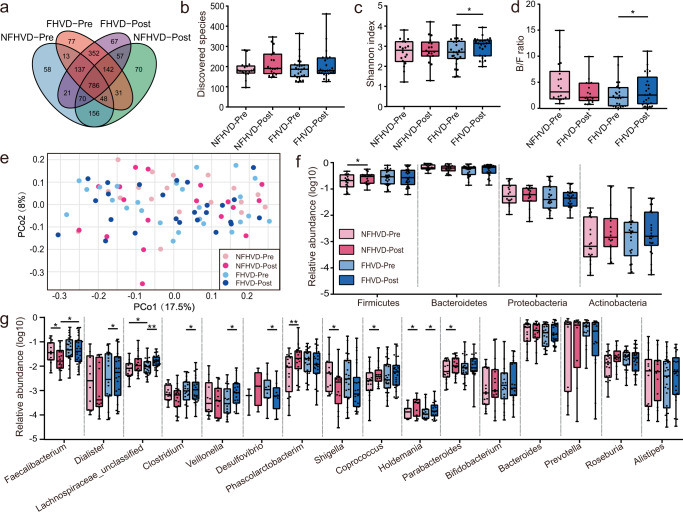
Gut microbial composition differences in FHVD and NFHVD. Variations in (**a**) Venn diagram of the overlap at OTU level. (**b**) The discovered species. (**c**) The alpha diversity (Shannon index). (**d**) The Bacteroidetes/Firmicutes ratio. (**e**) Overall gut microbial structure, principal co-ordinates analysis based on the Bray-Curtis distance. (**f**) The relative abundance on phylum level. (**g**) The relative abundance on genus level. FHVD-Pre (*n* = 25), FHVD-Post (*n* = 25), NFHVD-Pre (*n* = 18), and NFHVD-Post (*n* = 18). OTU, Operational Taxonomic Unit. Wilcoxon matched pair signed-rank tests (two tailed) were used to analyze pairwise Pre and Post within diets. A Mann-Whitney test was used to analyze differences between the FHVD and NFHVD groups at the same time point. *P < 0.05 and **P < 0.01. The bounds, whiskers and percentile of each box plot represented maximum, 75 percentile, median, 25 percentile and minimum from the top to the bottom respectively.

After 8 weeks of intervention, genomic DNA was extracted from each of 86 fecal samples for 16S rRNA gene sequencing of the V4 region to examine the gut microbiota. The Shannon curve indicated that the sequencing depth covered virtually the full range of the gut microorganisms (Supplementary Fig. 2). A total of 2085 OTUs were identified according to Fig. 1a, of which 58, 70, 77, and 67 OTUs were characteristic among NFHVD-Pre, NFHVD-Post, FHVD-Pre, and FHVD-Post, respectively. In addition, the FHVD influence on the amount and proportion of bacteria was assessed in individuals by the discovered species and Shannon index, as shown in Fig. 1b, c. The number of species in the two treatment groups were not significantly different, but there was a slight significant increase of Shannon index after FHVD intervention. Then, we assessed the gut microbiota composition through principal co-ordinate analysis over the unweighted UniFrac distance (Fig. 1e), none of the changed beta diversities were significantly different between groups during intervention (*P* > 0.05).

The taxa changes were further investigated at different classification levels horizontally and vertically in different cohorts. Specially, At the phylum level (Fig. 1f), the predominant taxa were *Firmicutes*, *Bacteroidetes*, *Proteobacteria*, and *Actinobacteria*. In particular, the relative abundance of *Firmicutes* showed a significant increase after NHFVD intervention (*P* = 0.0385), while other phyla showed no significant changes. As shown in Fig. 1d, the B/F ratio decreased after NFHVD intervention, with no significant difference (*P* = 0.0814), but significantly increased after the FHVD intervention (*P* = 0.0105).

At the genus level (Fig. 1g), there were 16 dominant genus taxa in healthy individuals. The high-abundance of *Bacteroides* and *Prevotella* were not obviously changed by FHVD intervention. *Faecalibacterium*, was significantly reduced with the NFHVD (*P* = 0.0268) but recovered with the FHVD by horizontal comparison (*P* = 0.0389). In addition, the abundances of *Dialister* (*P* = 0.0499), *Veillonella* (*P* = 0.0163), and an unclassified *Lachnospiraceae* organism (*P* = 0.0001) were increased specifically by the FHVD. Both diets increased the abundance of *Coprococcus* after intervention, but it only showed significance in the NFHVD (*P* = 0.0385). *Holdemania* was the only taxon that was significantly enriched by both the NFHVD and FHVD. In addition, *Shigella* and *Desulfovibrio* were found to had a similar reducing tendency in the FHVD, however, only the latter (*P* = 0.0163) showed significant difference.

### Co-occurrence network differences between diet interventions

**Fig. 2:**
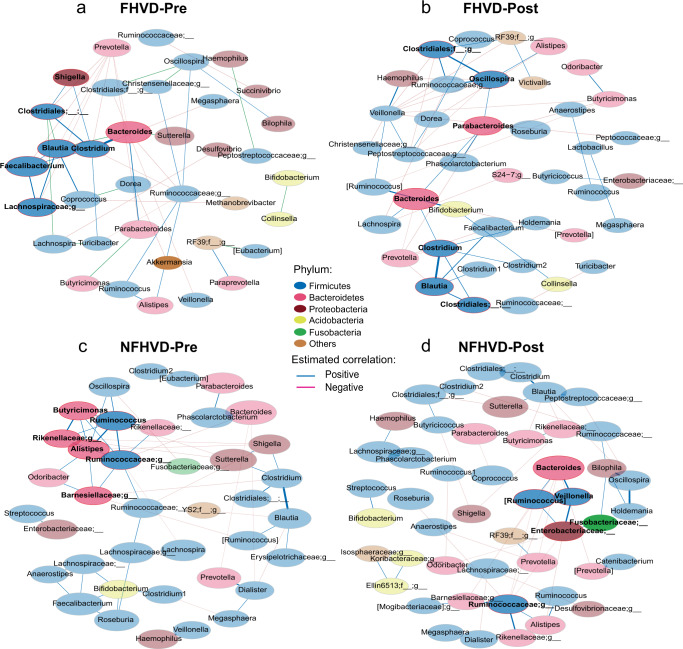
FHVD (*n* = 25) and NFHVD (*n* = 18) altered gut microbiome co-occurrence network. (**a**) FHVD-Pre, (**b**) FHVD-Post, (**c**) NFHVD-Pre, (**d**) NFHVD-Post. All networks were shown with each genus and co-occurrence relationship indicated by a node and links, core genus in each group was marked with deep color node and red edge.

Next, to investigate the patterns of interactions between gut microbial communities, the co-occurrence networks of the FHVD and NFHVD were constructed based on 16S rRNA gene sequencing data (Fig. 2). A total of 35 and 41 nodes were discovered in FHVD-Pre and -Post respectively, including 21 mutual nodes. For another, 39 and 49 nodes were found in NFHVD-Pre and -Post severally with 29 shared nodes in two groups. In addition, significant correlations including 28 positive and 16 negative correlations were revealed before the FHVD, while the number changed to 29 positive and 22 negative after the intervention. For the NFHVD, 35 positive and 16 negative correlations were revealed before the intervention, followed by 25 positive and 23 negative correlations after the NFHVD. Notably, the core taxa showed slight variation, with disappearance of *Shigella*, *Sutterella*, *Desulfovibrio*, and appearance of *Parabacteroides* in the FHVD.

### Metabolic function variation of gut microbiota in the *Ficus hirta Vahl* diet

**Fig. 3:**
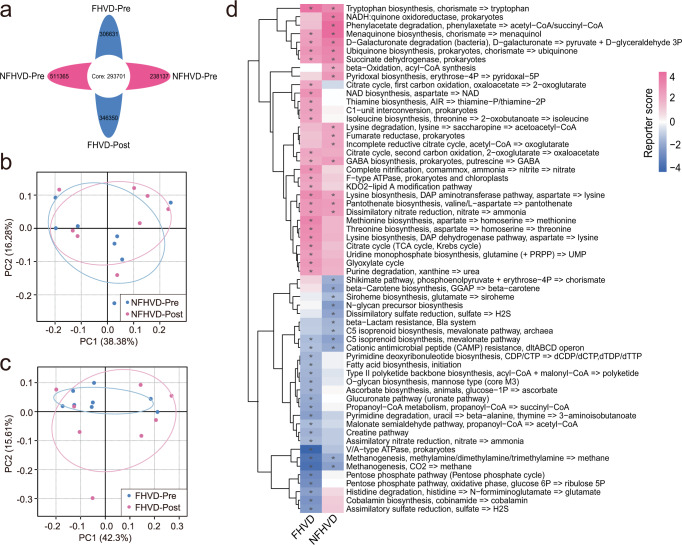
Comparison of gut microbial metagenomic functions between FHVD (*n* = 8) and NFHVD (*n* = 8). Changes in (**a**) Venn diagram of the gene counts. PCA (Principal Components Analysis) plot of (**b**) NFHVD and (**c**) FHVD based on Bray–Curtis distance according to KEGG Ontology (KO) profiles. (**d**) Microbial metabolic module alternation in FHVD and NFHVD. Asterisk denotes reporter score (RS) of module > 1.65 or < −1.65 were considered to be significant. Pink, enriched by after intervention; Blue, enriched before intervention.

More recently, MicroPITA was commonly applied to select specific samples from microbiome data to gain insight into the mechanism by which the gut microbiome may interact with the host^16,17^. Based on the 16S rRNA gene sequencing results of 86 samples, we selected 32 typical fecal samples (8 samples in each group) by supervised method to conduct metagenomic sequencing. In total, 293701 core genes were detected. 511365, 238137, 306631, and 346350 feature genes were observed in NFHVD-Pre, -Post, FHVD-Pre, and -Post, respectively (Fig. 3a). Principal component analysis (PCA) in different cohorts showed that the sample variation of the FHVD (Fig. 3c) was distinctly higher than that of the NFHVD (Fig. 3b). These genes belonged to 7256 KEGG Orthologs (KOs) and were distributed in 259 metabolic pathway modules. Furthermore, 69 KEGG pathway modules (34 enriched) showed significant differences in the FHVD, and 58 modules (29 enriched) were significantly changed by the NFHVD. The variation in the corresponding KEGG modules is displayed in Fig. 3d, and significance were shown according to the reporter score (|reporter score | > 1.65) of each module.

Particularly, The FHVD had a higher abundance of the synthesis pathways involving vitamin B1 (thiamine, RS = 1.932), B5 (pantothenate, RS = 2.313), and K2 (menaquinone, RS = 1.938), while the latter was only enriched by the NFHVD (RS = 3.843). Besides, NAD synthesis (RS = 2.463), citrate cycle (RS = 2.345), and the glyoxylate cycle (RS = 2.275) were enhanced by the FHVD. In contrast, the pentose-phosphate pathway was significantly decreased (RS = 3.194). The enhanced pathways from essential amino acids synthesis, including threonine (RS = 2.615), methionine (RS = 2.879), tryptophan (RS = 3.551), lysine (RS = 2.583), and isoleucine (RS = 1.840), was profiled in the FHVD, while only tryptophan (RS = 2.693) and lysine (RS = 1.843) were enriched by the NFHVD.

### Widely targeted metabolic profiles difference in the *Ficus hirta Vahl* diet

**Fig. 4:**
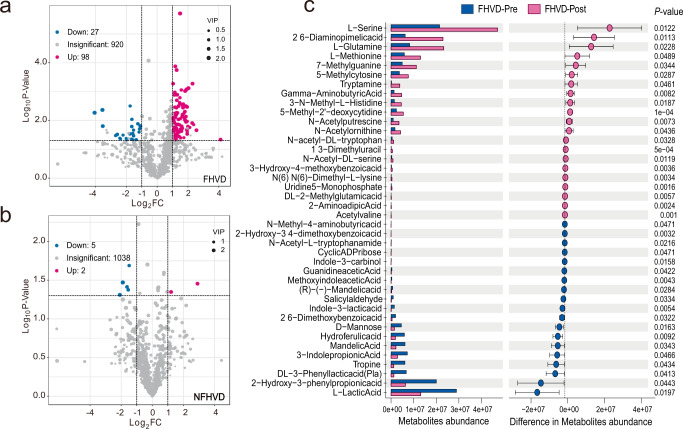
Comparison metabolite profiles differences by FHVD (*n* = 8) and NFHVD (*n* = 8) intervention. Volcano plot comparing metabolites in (**a**) FHVD and (**b**) NFHVD. (**c**) Differences of metabolite abundance and standard deviation in FHVD, the data were presented as Mean ± SD, significance of each metabolite was listed as *P*-value.

To further reveal the metabolic functional variation driven by the FHVD, widely targeted metabolic profiles were used to evaluate the effect of the FHVD on the host metabolite profiles from fecal samples. In this part, the variable importance in projection (VIP) scores were applied to estimate the enrichment or reduction in metabolites through intervention with the two diets. It was observed that the widely significant changes were enriched with 98 metabolites and downregulated with 27 metabolites by the FHVD, while a few changes were observed after the NFHVD: an increase in 2 metabolites and a reduction in 5 (Fig. 4a, b). The significant changes in the abundance of the main metabolites are shown in Fig. 4c. In particular, the FHVD mainly contributed to metabolites such as l-serine (*P* = 0.0122), l-methionine (*P* = 0.0489), gamma-aminobutyric acid (GABA, *P* = 0.0083), l-glutamine (*P* = 0.0228), and tryptamine (*P* = 0.0461). Other metabolites received little attention because of the fewer report with host health and gut microbiota.

### Concentration and gene abundance variation of short-chain fatty acid in the *Ficus hirta Vahl* diet

**Fig. 5:**
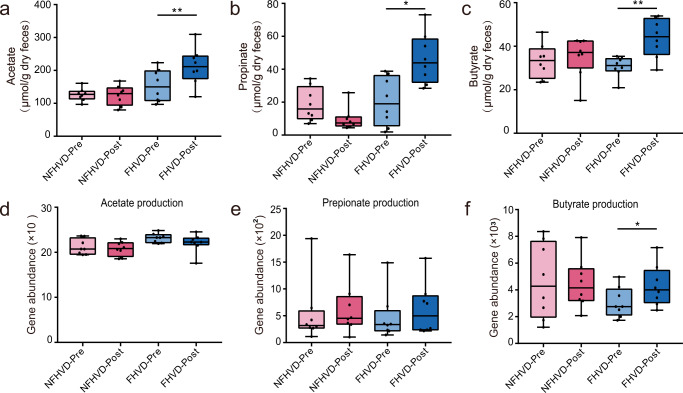
FHVD altered gut microbiota fermentation of carbohydrates to produce SCFAs. Changes in fecal concentrations of (**a**) acetate, (**b**) propionate, and (**c**) butyrate. Changes in the abundance of genes that encode the key enzymes in (**d**) acetate production [formate-tetrahydrofolate ligase *fhs* and acetate kinase *ack*], (**e**) propionate production [lactoyl-CoA dehydratase *lcd*, propionaldehyde dehydrogenase *pduP*, and methylmalonyl-CoA decarboxylase *mmd*], and (**f**) butyrate production [butyryl–coenzyme A (butyryl-CoA):acetate CoA transferase *but* and butyrate kinase *buk*]. The bounds, whiskers and percentile of each box plot represented maximum, 75 percentile, median, 25 percentile and minimum from the top to the bottom respectively.

Our study also showed clear relevant changes in a number of gut microbial fermentation metabolites. As shown in Fig. 5a–c, acetate (*P* = 0.0122), propionate (*P* = 0.0122), and butyrate (*P* = 0.0122) were distinctly improved by the FHVD but not by the NFHVD. Meanwhile, there was an increase in the key gene abundance of the butyrate synthesis pathway in the FHVD (*P* = 0.0391), although no significant changes were observed in acetate and propionate production (Figs. 5d–f). Next, the nutrient contents in water extract of FHV was measured Supplementary Fig. 3), including total sugar, protein, flavonoid, and polyphenol. The results showed that FHV provided a larger amount of carbohydrate than other components. In-vitro fermentation of FHV extract by the human gut microbiota also showed significant increases in acetate, propionate, and butyrate (Table 1).

**Table 1:** Concentrations of SCFAs in in-vitro incubation solutions at different time points.

SCFAs	Samples	Anaerobic incubation time (h)				
(mM)		0	3	6	12	24
Acetate	Control FHVD	0.31 ± 0.02	0.34 ± 0.10 a 2.35 ± 0.25 b	1.99 ± 0.40 a 2.69 ± 0.41 b	2.07 ± 0.28 a 9.13 ± 0.70 b	5.79 ± 0.11 a 6.40 ± 0.80 b
Propionate	Control FHVD	0.29 ± 0.02	0.51 ± 0.02 a 1.67 ± 0.15 b	1.36 ± 0.19 a 2.67 ± 0.27 b	1.26 ± 0.10 a 7.38 ± 0.46 b	3.40 ± 0.07 a 6.04 ± 0.57 b
Butyrate	Control FHVD	0.33 ± 0.01	0.42 ± 0.02 a 0.88 ± 0.01 b	0.92 ± 0.06 a 1.59 ± 0.08 b	1.25 ± 0.10 a 5.54 ± 0.25 b	2.41 ± 0.02 a 5.46 ± 0.45 b

Each data was conducted in triplicates, and the value was presented as Mean ± SD. Different lowercase letters indicate significant differences (*P* < 0.05) among different groups.

### Association analysis of Gut gut metagenome associated with feature metabolites in FHVD

**Fig. 6:**
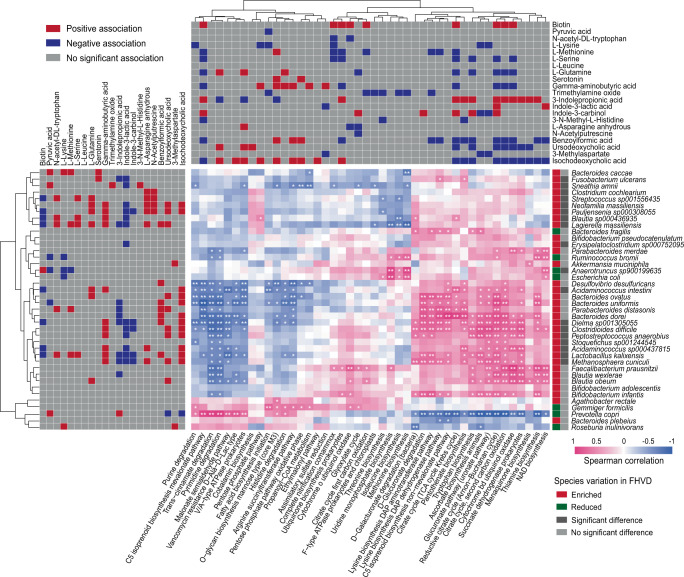
The tripartite correlation heatmap of gut microbial species in FHVD, KEGG pathways modules and fecal metabolites. The left panel denotes the Spearman correlations between species and fecal metabolites. The top panel denotes the Spearman correlations between pathway modules and fecal metabolites. The right panel denotes the significance of enriched or reduced species.

A total of 41 pathways were significantly correlated with 21 metabolites and 18 significantly enriched species, which highlighted the importance of microbial functions and taxa in interactions with fecal metabolites to affect host health (Fig. 6). For instance, biotin was positively correlated with the citrate cycle and coenzyme synthesis, while less significance was found in association with enriched species. Most of essential amino acids were positively correlated with FHVD-enriched species, such as *Streptococcus* spp., *Blautia* spp., and *Lactobacillus kalixensis*, while the latter was only significantly co-enriched with Methionine. Besides, GABA levels were positively associated with a wide range of enriched species, especially acid-production bacteria such as *Blautia* spp., *Lactobacillus* spp., and *Acidaminococcus* spp. Moreover, the citrate cycle and vitamin synthesis pathways exhibited a positive correlation with *Bacteroides dorei*, *Ruminococcus bromii*, and *Blautia obeum*, while few metabolites showed distinct correlation with these pathways.

## Discussion

Dietary intervention in vivo simulated food intake in normal human daily life with complex interactions between gut microbiota and hosts. It was concluded that the intake of nutrients was not significantly impacted by following an FHVD (Supplementary Table 3), indicating that the variation of gut microbial composition and function were mainly driven by additional FHV in daily diet. Besides, the FHVD improved the taxa diversity after 8 weeks of intervention (Fig. 1b), and the result of principal co-ordinate analysis over the unweighted UniFrac distance (Fig. 1e) showed that the entire diversity was mainly driven by individual differences, because there was no overlap between paired samples with the individuals clustering together. Thus, it was found that there were significant differences in the diversity between pre- and post-intervention with the FHVD (horizontal and vertical), but interindividual variation seemed to contribute more to horizontal differences, leading to no significant variation on blood biochemicals (Supplementary Table 4).

It was also obvious that the overall microbial structure showed significant alterations by the FHVD and NFHVD, however, their variation tendency was distinctly different. Although fewer significant change of *Bacteroidetes* was found during treatments, it was worth noting that the decrease of *Bacteroidetes* during the NFHVD and increase during the FHVD might jointly impact the *Bacteroidetes*/*Firmicutes* ratio (B/F ratio). Recently, a large-scale population study of the gut microbiota revealed that most OTUs from *Bacteroidetes* were more prevalent among healthy individuals in Guangdong Province^18^. In addition, as the report described, a higher B/F ratio was related to decreased obesity risk and maintaining gut homeostasis^19^; our results suggested that the additional FHV in the diet had the potential of healthcare by altering the gut microbiota composition. Moreover, the enterotype-determined *Bacteroides* and *Prevotella* were not significantly changed by FHVD intervention, suggesting that neither of the two medium diets could influence the individual enterotypes, while some low abundance clusters showed noteworthy differences. In the FHVD, the enriched *Dialister* spp. and *Veillonella* spp. are recognized to be propionate producers through the succinate pathway^20^. Meanwhile, some species from *Lachnospiraceae* are known to utilize acetate and lactate to produce butyrate^21^. Another butyrate producer, *Coprococcus* spp., was enriched in both diets, but it only showed significance in the NFHVD.

*Faecalibacterium prausnitzii*, a species from the *Faecalibacterium* genus which was listed as a live biotherapeutics^22^, is beneficial to human health by generating immunoregulatory molecules such as butyrate^23^ and negatively connected with a variety of diseases, such as T2DM^24^, colitis^25^, and gout^26^. Interestingly, *Faecalibacterium* was dramatically reversed in FHVD-Post (compared with NFHVD-Post) (*P* < 0.05), suggesting a preservation of the probiotics by the FHVD. For the reducing taxa, genera from *Proteobacteria*, such as *Shigella* and *Desulfovibrio*, have been considered to contain opportunistic pathogens and proinflammatory bacteria^27,28^. Simulated results were also found in an in-vitro fermentation system of prebiotics by the gut microbiota^29^. Overall, it was suggested that the addition of FHV enriched the abundance of *Faecalibacterium* and SCFA-producing bacteria and inhibited potential pathogens, thereby protecting the gut microbiome in healthy individuals.

Obviously, The gut microbiome is an ecosystem with complex interactions. It was suggested that microbial interactions are promoted in both diets (Fig. 2). Notably, the core taxa showed slight variation, with disappearing *Shigella*, a potential pathogen in *Proteobacterium*^28^. Comparable results were also found in *Sutterella* and *Desulfovibrio*. The results indicated that the relative abundance and correlation of pathogenic bacteria were restrained by the FHVD. Meanwhile, *Parabacteroides* appeared to be a core node after the FHVD, which was reported to play an opposing role with diverse conditions in recent research^30^. With the NFHVD intervention, the decrease in the positive correlation and increase in the negative correlation suggested that the microbial structure tended to be enriched in specific taxa, although the topological nodes showed an obvious improvement. Moreover, *Faecalibacterium* and *Blautia* had a positive correlation with the FHVD-Pre and -Post networks, reflecting a possible synergistic relationship between beneficial bacteria. A positive correlation was also found between *Clostridium* and *Blautia*. However, the underlying mechanism remains to be revealed because the functions of taxa in *Clostridium* are complex^31,32^. In summary, these phenomena indicate that gut microbiota co-occurrence networks are structured to be healthier by an FHVD.

Furthermore, the effect of the FHVD on gut microbial metabolism showed distinct diverse in the two diets. In particular, the synthesis pathway of B vitamins were found specifically enriched in the FHVD. Previous studies have emphasized the importance of microbial water- and fat-soluble vitamins in modulating the gut microbiota and host immunoreactions in disease^33,34^. Besides, The FHVD seems to modulate energy metabolism via strengthening the citrate cycle. Usually, the citrate cycle occurs in the mitochondrial matrix and is a core integration center for the carbohydrate, lipid, and protein metabolic pathways. As a critical component of the citrate cycle, citric acid exerts antioxidant, antiapoptotic, and anti-inflammatory actions in the liver, brain, and cardiac tissues^35^. The decrease in the sulfate reduction pathway was enhanced by the FHVD compared with the NFHVD, as the major product of this pathway, and it was reported that the effect of H_2_S on health was complex^36^, further studies will need to assess with clinical phenotypes. Moreover, despite the same partial trends in the NFHVD, a global enrichment pathways of amino acid synthesis was found with the FHVD, such as threonine, methionine, and isoleucine, there is an increasing body of evidence indicating that the synthesis and metabolism of amino acids are crucial pathway to modulate host physiology. Amino acids can be synthesized by gut bacteria and are released into the intestinal tract for further entry into the circulatory system or utilized for the synthesis of bacterial cell components and functional metabolites such as SCFAs^37^, meanwhile, they could further alter energy homeostasis, nutrition metabolism, gut health, and immunity^38^. Other metabolic pathways also showed dispersive enrichment by intervention with the FHVD. However, most of them were less frequently reported to be connected with host physiology.

Widely targeted metabolic assay was used to evaluate the effect of the FHVD on the fecal metabolite profiles. Previous study reported that serine in the gut improved colonic morphology and alleviated inflammatory responses in mice with colitis^39^. Glutamine, one of the most abundant amino acids in the human body, protects gut health by repairing intestinal barrier function^40^. However, little evidence has been obtained to confirm whether these enriched metabolites were originated from the gut microbiota. In recent decades, tryptophan metabolites have been verified to be closely connected with human health by regulating neurotransmission and cytokine signaling^41^. Tryptamine is mainly transferred from tryptophan by decarboxylases encoded in a variety of *Firmicutes* genomes, such as *Blautia* spp., *Ruminococcus* spp., *Clostridium* spp., and *Lachnospiraceae* spp^42^., and the latter pair was found to be significantly enriched in an FHVD. 5-Hydroxy tryptamine, another health-related tryptophan metabolite, was found to increase 1.38-fold, although the difference was not significant. Additionally, endogenous GABA was also considered to be a regulator of the nervous system through the gut-brain axis^43^. The enhancement of neurotransmitter-benefit metabolites might be related to the function of the effect of supplying Qi and reinforcing deficiencies of FHV^4^. However, more evidence is needed.

A consensus has been reached that beneficial bacteria can produce SCFAs, which lower the gut pH and inhibit the growth of pathogenic bacteria^44^. In addition, SCFAs are also signal microbial metabolites to regulate host immunoreactions through G protein-coupled receptors (GPCRs) and fatty acid receptors (FFARs)^45^. In our study, the dry weight of three predominate SCFAs in the feces, including acetate, propionate, and butyrate were promoted by the FHVD. There are two major pathways by which exogenous herbal nutrients of promote SCFA levels in the gut^46^, one of which provides a fermentable carbon source that can only be metabolized by the gut microbiota. We considered that the most abundant carbohydrate could sever as available substrate for the fermentation of gut microbiota, and the results of in-vitro fermentation also confirmed the contribution of FHV extract on SCFAs. Our subsequent study will focus on the further functions of saccharides derived from FHV. In fact, by concluding the key enzyme gene abundance of the synthesis pathway of above SCFAs, the gene abundance of the butyrate synthesis pathway was found significantly increased in the FHVD. On the other hand, Acetate- and propionate-producing genera, including *Lachnospiraceae* and *Dialister*, were enriched in the FHVD, and the increased SCFA levels could also be related to enzyme activity in variety taxa^46^.

Given the major metabolite differences driven by the FHVD, tripartite correlation analysis was performed to demonstrate the relationship between microbial pathway modules, taxa and metabolites (Fig. 6). The positive correlation between essential amino acids and FHVD-enriched *Streptococcus* spp., *Blautia* spp., and *Lactobacillus kalixensis*, rather than functional pathway, suggested that the increased amino acids were likely driven by interaction of these species. Additionally, GABA levels were positively associated with a wide range of enriched species, especially acid-production bacteria such as *Blautia* spp., *Acidaminococcus* spp. and *Lactobacillus kalixensis*. Another opinion is that GABA could be observed only in an acidic environment (pH < 5.5) in vitro^47^, suggesting that an FHVD might promote these species to improve GABA concentrations by enhancing the SCFAs synthesis pathway. Despite the citrate cycle and vitamin synthesis pathways were co-enriched with several indistinctive and enriched bacteria (*Bacteroides dorei*, *Ruminococcus bromii*, and *Blautia obeum*), but few metabolites showed a distinct correlation with these pathways. This implied that the influence of an FHVD on the fecal metabolite profiles was more likely through alterations in the gut microbial consortium to modulate pathway abundance indirectly rather than individual species.

Overall, our results emphasized the clear microbial taxa variation with an FHVD and its close association with metagenomic functions and fecal metabolic profiles, providing a new understanding of the role of dietary FHV in modulating host physiology. Current evidences verified that the healthcare function of FHVD might be associated with enhancing the connection of potential benefited bacteria and health-promoted metabolites. Future studies in larger clinical trial cohorts and animal experiments still need to be conducted to obtain a better understanding of the relationship between FHV components and gut microbiota in specific disease phenotypes.

## Methods

### Study design

The dried root of FHV was purchased from Jinyuan Green Life Co., Ltd. (Heyuan, China). The FHVD and NFHVD were prepared in the form of soup according to the Cantonese recipe shown in Supplementary Table 1. The control group of NFHVD contained no FHV and was prepared the same as FHVD to reveal the effect of the additive FHV. In total, 48 healthy participants (25 men and 23 women) were recruited for this research; no participants had hyperglycemia, hyperlipemia or gastrointestinal disease; individuals with a history of antibiotic use within 6 months were also excluded from this research. All participants were randomly allocated to receive FHVD (*n* = 29,) or NFHVD (*n* = 19). There were no significant differences (*P* > 0.05) in participant physical signs, as summarized in Supplementary Table 2. The diet intervention process was presented in Supplementary Fig. 1. After passing through a two-week baseline measurement (week 0), the participants entered an 8-week intervention to receive 250 ml of soup containing FHVD or NFHVD per day at a frequency of random four times at workday per week. Individuals who failed to complete the diet intervention were excluded from the analysis. Fecal samples were collected before (week 0, for the Pre-treatment assessment) and after (week 8, for the post-treatment analysis) diet intervention for further analysis. Finally, 5 individuals were excluded from the analysis due to protocol violations (3 individuals) and unfinished food records (2 individuals). A total of 43 individuals and 86 16S rRNA gene sequencing samples were included in the analysis to assess the effects of FHVD on the gut microbiota composition.

Written informed consents were taken from all participants before enrollment. Ethics approval was accepted by the Ethics Committee of The First Affiliated Hospital of Guangzhou University of Chinese Medicine (Guangzhou, China), document NO. ZYYECK2019-032-XZ-01, ChiCTR Regis-tration NO. ChiCTR2200056956.

### Dietary intake assessment

During the intervention period, participants were required to repast at a specific dining hall to normalize their dietary nutritional intake. Briefly, the participants were given information on the food nutrient content, and they were instructed to finish a continuous three-day food record at sampling weeks. Then, the average ingestion of macronutrients was calculated and checked for completeness. The intake of macronutrients between -Pre and -Post in the two diets was compared to assess dietary balance.

### Fecal sample collection and pretreatment

Fecal samples were collected individually following an SOP of self-collection (http://www.microbiome‐standards.org), and a sampling kit with stabilizing solution was obtained from Beijing Genomics Institute (BGI). Samples were sent to the laboratory within two hours and immediately stored at −80 °C for further sequencing at BGI and lyophilized for metabolic analyses at Metware Biotechnology Co., Ltd. (Wuhan, China).

### 16S rRNA gene sequencing

Bacterial genomic DNA was used as a template to amplify the V4 hypervariable region of the 16S rRNA gene with the forward primer 515 F (5′-GTGCCAGCMGCCGCGGTAA-3′) and the reverse primer 806 R (5′-GGACTACHVGGGTWTCTAAT-3′). Validated libraries were used for sequencing on the Illumina HiSeq 2500 platform and generated 2 × 250 bp paired-end reads. The quality control and clustering results were analyzed by deblur method in QIIME 2.0 software (version 2019.7). Optimized sequences were clustered at a similarity of 97% and blasted with the Green Gene Database to identify the taxa. Further analyses, such as alpha diversity, beta diversity, and taxonomic distinctness, were conducted in R (version 3.5.2) using the in-house script of the vegan package (version 3.3.1).

### Metagenomic sequencing

To further discern the role of FHVD in modulating gut microbiome functions, microPITA (microbiomes Picking Interesting Taxonomic Abundance) was applied to select typical samples of each group for metagenomic sequencing^48^. A total of 32 core samples were selected by supervised method^48^, with every 8 samples in FHVD-Pre, FHVD-Post, NFHVD-Pre, and NFHVD-Post respectively. Genomic DNA fragmentation and library construction were conducted at BGI, and metagenomic sequencing was performed on the MGISEQ-2000 platform at a depth of 10 GB. Analysis methods and the calculation of gene abundance and Report Score were performed according to previous studies^26^.

### Widely targeted metabolomics detection

Widely targeted metabolomics detection was performed to analyse the global fecal metabolites. In brief, a 20 mg lyophilized faeces was mixed with 400 μL of 70% methanol-water (internal standard extractant), vortexed for 3 minutes and sonicated for 10 minutes in an ice water bath. Then, the mixture was centrifuged (12000 rpm, 4 °C) for 10 min, and the supernatant was collected for analysis.

The sample extracts were analyzed using an LC–ESI–MS/MS system (UPLC, ExionLC AD; MS, QTRAP® System). The analytical conditions were as follows: the UPLC column was a Waters ACQUITY UPLC HSS T3 C18 column (1.8 μm, 2.1 mm×100 mm); the column temperature was 40 °C; the flow rate was 0.4 mL/min; the injection volume was 2 μL; the solvent system was water (0.1% formic acid):acetonitrile (0.1% formic acid); gradient program, 95:5 V/V at 0 min, 10:90 V/V at 11.0 min, 10:90 V/V at 12.0 min, 95:5 V/V at 12.1 min, 95:5 V/V at 14.0 min. Another column was also used for separation: UPLC column, Waters ACQUITY UPLC BEH Amide (1.7 μm, 2.1 mm × 100 mm); column temperature, 40 °C; flow rate, 0.4 mL/min; injection volume, 2 μL; solvent system, water (25 mM ammonium formate/0.4% ammonia):acetonitrile; gradient program, 10:90 V/V at 0 min, 40:60 V/V at 9.0 min, 60:40 V/V at 10.0 min, 60:40 V/V at 11.0 min, 10:90 V/V at 11.1 min, 10:90 V/V at 15.0 min.

The ESI source operation parameters were as follows: source temperature 500 °C; ion spray voltage 5500 V (positive), −4500 V (negative); ion source gas I, gas II, and curtain gas were set at 55, 60, and 25.0 psi, respectively. The results were compared with the internal database of Metware Biotechnology Co., Ltd. (Wuhan, China).

### Content analysis of components in FHV water extract

The water extract of FHV was prepared by following method: 200 ml water was added to 20 g FHV powder and incubated in boiling water for 2 h. Then, the extracting solution was concentrated in vacuum to 50 ml for components analysis. The mass concentrations of total sugar, protein, flavonoid, and polyphenol were measured according to previous studies^49,50^.

### Gut microbiota fermentation in vitro

Fecal samples for in-vitro incubation were collected from 4 random participants. The pretreatment of fecal slurry and incubation medium were prepared according to a previous study^51^. A total of 5 mL water extract containing 2 g FHV dried root was added to BNM medium and set as the FHVE group. Five milliliters of distilled water were added and set as the control group. Then, one milliliter of the fecal slurry was inoculated in an anaerobic bottle with 25 mL of BNM broth, and each group was inoculated in triplicate. Incubation was carried out under anaerobic conditions at 37 °C, and samples from 0, 3, 6, 12, and 24 h were collected for SCFA tests.

### SCFA detection

Sample pretreatment: 100 mg dried fecal powder was resuspended in 1 mL of distilled water and vortexed for 2 min. Then, 600 μL of the supernatant of the fecal slurry or fermentation broth was acidified with 20% (v/v) H_2_SO_4_. After vortexing for 1 min, 500 μL of n-butanol was added to the mixture and vortexed for 2 min. The supernatant was filtered through a 0.22 μm filter membrane for sample injection. An Agilent 7820 A gas chromatography system (Agilent Technologies, Santa Clara, CA) equipped with a flame ionization detector (FID) and a DB-FFAP capillary column (Agilent, 30 m × 0.25 mm × 0.25 μm) was used for component separation. The operating temperature conditions were set according to our previous report^29^. Each experiment was performed in triplicate.

### Statistical analysis

All statistical analyses were conducted with GraphPad Prism 7.0 software, and Student’s t test was used to analyze the in-vitro fermentation data. Paired microbiome, metagenomics and metabolomics data were analyzed by a nonparametric Wilcoxon matched-pairs signed-rank test, while the nonpaired data of different interventions were analyzed by the nonparametric Mann–Whitney test comparing ranks. The Spearman correlation was calculated to search for relations between the microbiome or gene abundance and the biomarker levels. *P* < 0.05 was regarded as a statistically significant difference.

## Supplementary information

Supplementary information

## Data Availability

The 16S rRNA gene and metagenomic sequencing data that support the findings of this study are available for public in Genebank database with accession numbers PRJNA804374 and PRJNA812687 respectively. The metabolome profile data and other details were list within the article and supplementary materials.
