# Supplementary material for: Association of human gut microbiota composition and metabolic functions with Ficus hirta Vahl dietary supplementation
Source: NPJ Sci Food. 2022 Sep 27;6:45. doi: 10.1038/s41538-022-00161-3 (PMC9515076; doi:10.1038/s41538-022-00161-3)
Supplement: Supplementary file 1 — Supplementary information [file 41538_2022_161_MOESM1_ESM.pdf]

Association of human gut microbiota composition and metabolic functions with *Ficus hirta Vahl* dietary supplementation

Ruiming Xiao [ab], Guangjuan Luo [ab], Wanci Liao [ab], Shuting Chen [ab], Shuangyan Han [ab], Shuli Liang [ab], Ying Lin [ab*]

[a]South China University of Technology South China Univ Technol, School of Biology & Biological Engineering,

[b]Guangdong Key Lab Fermentation & Enzyme Engineering, Guangzhou 510006, Peoples R China.

*Corresponding author: Ying Lin, South China Univ Technol, Sch Biol & Biol Engn, Guangdong Key Lab Fermentat & Enzyme Engn, Guangzhou 510006, Peoples R China

E-mail: feylin@scut.edu.cn

Supplementary Table 1. Daily ingredients formula in FHVD (n=25) and NFHVD (n=18) for each people.

Ingredient names	Food weight (g)		P value
	FHV Diet	NFHV Diet	
Pork chops	25.08 ± 0.29	24.79 ± 0.50	0.433
Maize	13.33 ± 0.33	13.14 ± 0.34	0.535
Carrot	13.63 ± 0.52	13.58 ± 0.40	0.902
FHV dried root	14.95 ± 0.28	0	< 0.001

Supplementary Note 1: The pork chops, maize, and carrot were cut into 2-3 cm of length, the dried root of FHV was cut into 0.2-0.4 cm of slice. Then, all ingredients were mixed and stewed in boiling water for 2 h. After cooking, the ingredients were abandoned and the 250 mL of soup was collected for each participant. The data were presented as Mean ± Standard deviation (SD) and analyzed by Student's t test, $P<0.05$ was regarded as a significant difference.

Supplementary Table 2. Characteristics of participants in FHVD (n=25) and NFHVD (n=18) at baseline.

	FHV Diet	NFHV Diet	P value
Age (years)	23.68 ± 2.84	23.00 ± 2.81	0.423
BMI (kg/m^2)	21.15 ± 2.13	20.87 ± 2.29	0.506
Urea (mmol/L)	4.93 ± 1.23	4.96 ± 1.21	0.956
CRE (μmol/L)	75.04 ± 15.47	79.55 ± 24.37	0.356
UA (μmol/L)	334.82 ± 107.24	369.49 ± 117.34	0.360

BG (mmol/L)	5.14 ± 0.38	5.23 ± 0.41	0.487
TG (mmol/L)	0.81 ± 0.24	0.86 ± 0.35	0.648
TC (mmol/L)	4.50 ± 0.71	4.51 ± 0.72	0.971
Male/Female	0.92	1.25	

Supplementary Note 2: BMI: Body mass index; CRE: Creatinine; UA: Uric acid; BG: Blood Glucose; TG: Total triglycerides; TC: Total cholesterol. The data were presented as Mean ± Standard deviation (SD) and analyzed by Student's t test, *P*<0.05 was regarded as a significant difference.

Supplementary Table 3. Macronutrients intake comparation of participants in FHVD (n=25) and NFHVD (n=18) at sampling week.

Nutrient	FHVD			NFHVD		
	Pre	Post	*P* value	Pre	Post	*P* value
Energy (kj)	1263.73 ± 432.60	1230.94 ± 396.72	0.290	1282.11 ± 270.03	1258.25 ± 195.35	0.468
Protein (g)	56.24 ± 22.84	51.99 ± 19.31	0.143	53.76 ± 14.62	50.57 ± 8.14	0.347
Carbohydrate (g)	186.84 ± 66.03	181.87 ± 72.76	0.375	201.15 ± 62.11	203.88 ± 43.24	0.442
Fat (g)	31.90 ± 16.11	31.23 ± 16.24	0.989	26.68 ± 10.99	24.69 ± 9.43	0.246
Diet fiber (g)	6.46 ± 2.61	5.61 ± 2.14	0.726	5.02 ± 2.03	4.91 ± 1.72	0.152

Supplementary Note 3: The data were presented as Mean ± Standard deviation (SD) and analyzed by Student's t test, *P*<0.05 was regarded as a significant difference.

Supplementary Table 4. Blood biochemicals of participants in a preliminary experiment of FHVD intervention (n=9).

	FHVD-Pre	FHVD-Post	*P* value
Urea (mmol/L)	5.28 ± 1.66	5.01 ± 2.11	0.516
CRE (μmol/L)	64.00 ± 14.82	66.00 ± 20.37	0.844
UA (μmol/L)	362.42 ± 99.65	359.77 ± 103.57	0.999
BG (mmol/L)	5.40 ± 0.24	5.45 ± 0.30	0.734
TG (mmol/L)	0.74 ± 0.18	0.80 ± 0.32	0.531
TC (mmol/L)	4.80 ± 0.84	4.69 ± 0.60	0.523
Urea/CRE	0.09 ± 0.05	0.09 ± 0.06	0.984

Supplementary Note 4: CRE: Creatinine; UA: Uric acid; BG: Blood Glucose; TG: Total triglycerides; TC: Total cholesterol. The data were presented as Mean ± Standard deviation (SD) and analyzed by Student's t test, $P<0.05$ was regarded as a significant difference.

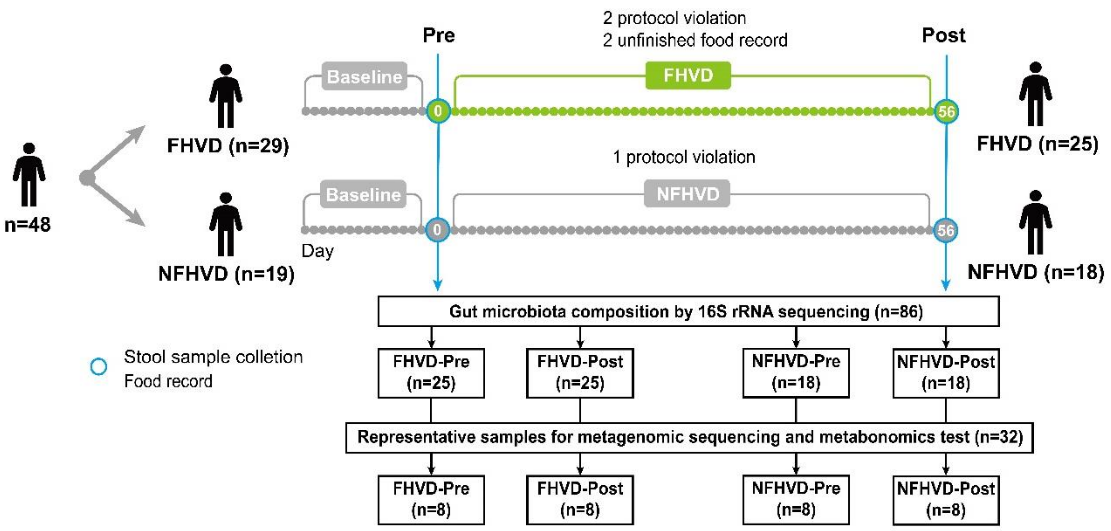

Supplementary Figure 1. Study design and flow diagram of data collection: Timeline of diet intervention, a two-weeks Run in period was set for stabilization and followed by 8-weeks intervention, fecal samples and corresponding food records was collected before and after intervention; By excluding unfitted samples, a total of 86 samples in FHVD-Pre (n=25), FHVD-Post (n=25), NFHVD-Pre (n=18), and NFHVD-Post (n=18) was involved to analyze gut microbial composition by 16S rRNA sequencing; MicroPITA was used to selected eight representative samples in each group for further metagenomic sequencing and metabolomic test. FHVD, *Ficus hirta Vahl* diet; NFHVD, non- *Ficus hirta Vahl* diet; Pre, before intervention; Post, after intervention; MicroPITA, microbiomes Picking Interesting Taxonomic Abundance.

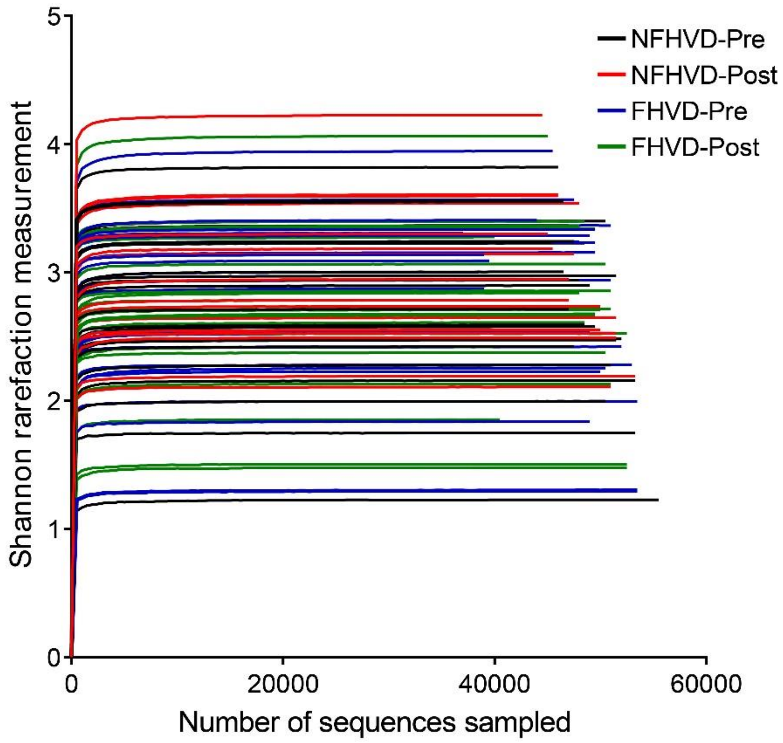

Supplementary Figure 2. Shannon rarefaction curve of each sample in FHVD and NFHVD.

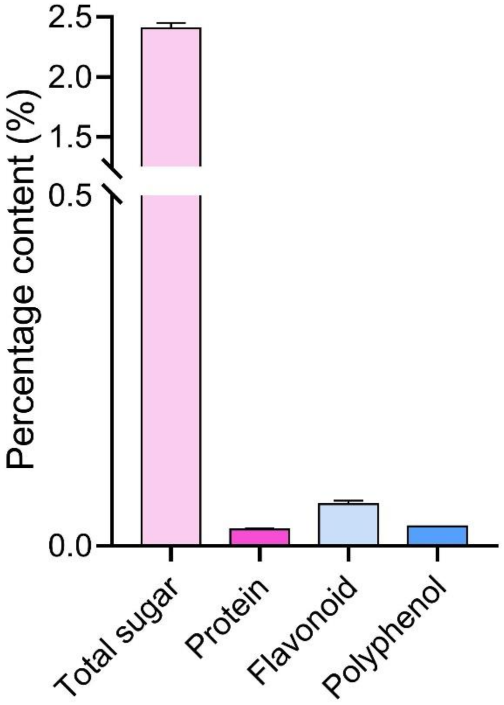

Supplementary Figure 3. Percentage content of Components in FHV water extract. The data were presented as Mean ± SD.
